# Comprehensive interventions to reduce occupational hazards among medical staff in the pathology department of five primary hospitals

**DOI:** 10.1186/s12889-023-16948-2

**Published:** 2023-10-31

**Authors:** Hui Wang, Dao Feng, Yong He, Xunhua Jin, Shui Fu

**Affiliations:** 1 Department of Pathology, The First People’s Hospital of Fuyang Hangzhou, Hangzhou, Zhejiang Province 311400 People’s Republic of China; 2 Department of Gynecology, First People’s Hospital of Linping District, Hangzhou, Zhejiang Province 311100 People’s Republic of China; 3 Department of Clinical Laboratory, Fuyang District Center for Disease Control and Prevention, Hangzhou, Zhejiang Province 311400 People’s Republic of China; 4 Hospital Sense Department, The First People’s Hospital of Fuyang Hangzhou, Hangzhou, Zhejiang Province 311400 People’s Republic of China; 5 Department of Clinical Laboratory, First People’s Hospital of Linping District, Hangzhou, Zhejiang Province 311100 People’s Republic of China

**Keywords:** Pathology department, Occupational hazard, Formaldehyde, Benzene, Carcinogenic risk, Noncarcinogenic risk

## Abstract

**Objective:**

To explore comprehensive interventions to reduce occupational hazards among medical staff in the pathology department of five primary hospitals.

**Methods:**

The indoor air quality in the pathology department of five primary hospitals and the health status of staff were investigated and analyzed. Formaldehyde and benzene concentrations in the technical and diagnostic rooms of the pathology departments were analyzed before and after comprehensive interventions. The Environmental Protection Agency risk assessment paradigm was used to assess the health risks from occupational exposure to benzene and formaldehyde. Consequently, considering the local environment, targeted comprehensive intervention measures were developed, including optimizing management, raising awareness, updating equipment, and replacing reagents.

**Results:**

Eye discomfort was higher among technicians in the pathology department than among clinical medical staff (*P* < 0.05). Before comprehensive interventions, formaldehyde concentrations were higher in the technical room than in the diagnostic room at the five primary hospitals (*P* < 0.05). However, compared to before interventions, formaldehyde and benzene concentrations in both rooms were significantly lower after comprehensive interventions. Furthermore, although medium risks of occupational exposure to benzene and formaldehyde remained in both rooms before and after comprehensive interventions, the risk values before interventions were higher than after comprehensive interventions. The staff of the technical rooms showed higher risk values that those of the diagnostic rooms before and after comprehensive interventions. Similarly, although hazard quotient (HQ) values for occupational exposure to benzene and formaldehyde were < 1 in both the technical and diagnostic rooms before and after comprehensive interventions, with lower noncarcinogenic risks, the values were higher before than after comprehensive interventions. Moreover, staff in the technical room had higher HQ values before and after comprehensive interventions than those in the diagnostic room. The use of environmentally friendly reagents for the preparation of frozen sections was effective.

**Conclusion:**

Comprehensive interventions significantly reduced occupational hazards among staff at the pathology department of five primary hospitals, which is of great practical significance to protect the health of staff.

**Supplementary Information:**

The online version contains supplementary material available at 10.1186/s12889-023-16948-2.

## Introduction

Formaldehyde and benzene play crucial roles as organic solvents in the fixation and preparation of pathological specimens. However, due to the pronounced toxicity associated with benzene, less toxic alternatives like toluene and xylene are frequently employed as substitutes for benzene in fixatives. Nevertheless, toluene and xylene may sometimes contain trace amounts of benzene. According to the classification of the International Agency for Research on Cancer (IARC), formaldehyde and benzene have been identified as human carcinogens [1]. Numerous studies have demonstrated that in addition to their association with cancer and leukemia, formaldehyde and benzene exposure can also lead to the development of cardiovascular and nervous system diseases, as well as respiratory symptoms [2, 3]. Because of their high volatility, formaldehyde and benzene are regarded as occupational indoor air pollutants. The evaporation of these substances in hospitals, clinics, and academic laboratories can potentially result in exposure for laboratory technicians and other personnel working in these environments. Research has shown that medical staff who are exposed to formaldehyde in hospital laboratories are at greater risk than others who work in other workplaces, since they are directly exposed to higher-concentration formaldehyde [4, 5] through inhalation or the skin. People who are exposed to benzene mainly absorb benzene through the respiratory system. In addition, when workers are exposed to benzene vapor, percutaneous absorption through the skin also becomes a significant route of exposure [6]. Therefore, it is necessary to assess the health risk of technicians who are often exposed to formaldehyde and benzene in pathology departments. The health risk assessment is a qualitative or quantitative assessment of occupational harmful factors at workplaces, with the aim of estimating the potential health damage caused by occupational harmful factors and the possibility of health damage. Health risk assessment can provide a scientific basis for predicting the long-term effects of occupational harmful factors and for formulating prevention and control strategies.

The inhalation risk assessment model of U.S. Environmental Protection Agency (EPA), the model of Singapore Ministry of Manpower (MOM), the model of the International Council of Mines and Metals (ICMM), and the technical guide for health risk assessment in Chemical Exposure Environment (WS/T 777–2021) are the four methods commonly used for health risk assessment [7–9]. The model of the Singapore Ministry of Manpower (MOM) and the ICMM model are used for semi-quantitative risk assessment based on hazard level data, dose–response relationship and exposure level, while the inhalation risk assessment model of U.S. Environmental Protection Agency (EPA) and the technical guide for health risk assessment in Chemical Exposure Environment (WS/T 777–2021) are quantitative risk assessments available for assessing the health risks of chemical poisons in various industries. In the context of formaldehyde and benzene risk assessment, the EPA (Environmental Protection Agency) method is extensively adopted in research. This method involves evaluating the cancer risks associated with formaldehyde exposure by estimating exposure concentration (EC) and lifetime cancer risk (LCR). Additionally, the hazard quotient (HQ) is calculated to quantify risks in biological matrices [10, 11]. In a preliminary investigation of the pathology department of the local hospitals, the present authors concluded that formaldehyde and benzene-containing chemical reagents are used extensively for anticorrosion and the preparation of pathological slices. As such, pathological technicians are exposed to potential risks. The EPA model was used to analyze the health risks of pathological technicians, and scientific measures were adopted to meet the requirements for occupational exposure limits.

In addition, most studies have reported on the concentration of important chemical reagents such as formaldehyde and benzene [12, 13]; however, studies comprehensively evaluating the exposure time, which may lead to biased results, are scarce. The Environmental Protection Agency (EPA)’s risk assessment paradigm [7] has been used since the 1880s to determine the possibility and severity of occupational hazards. Therefore, the present study evaluated the carcinogenic and hazard risks of formaldehyde and benzene exposure among staff based on the concentration level and exposure time using the EPA risk assessment paradigm [14, 15]. In addition, the present study formulated comprehensive interventions for occupational hazards in the pathology department based on two perspectives of both concentration level and exposure time. Practical and feasible methods using carcinogenic and hazard risk coefficients and the health status of medical staff were also explored to provide a standardization and normalization model.

## Materials and methods

### Study subjects

Overall, we included 22 medical technicians (They followed established procedures to perform tasks such as tissue dehydration, embedding, sectioning, and staining, ultimately producing qualified pathological slides. Additionally, they conducted basic tests related to immunohistochemistry and molecular biology. Furthermore, there were 24 non-pathological professional medical staff from the medical examination center. These medical examiners can be divided into different specialties, including 6 internal medicine examiners, 6 surgical examiners, 6 gynecological examiners, and 6 facial features examiners) from the sampling, technical, and diagnostic rooms of the pathology department of five hospitals—The First People’s Hospital of Fuyang Hangzhou (hereafter referred to as “First District Hospital”), The Second People’s Hospital of Fuyang Hangzhou (“Second District Hospital”), Materal and Child Health Care Hospital of Fuyang Hangzhou (“District MCH Hospital”), Hangzhou Fuyang District Hospital of TCM (TCM) Hospital (“District TCM Hospital”), and Hangzhou City Fuyang TCM Orthopedics Hospital (“District Orthopedic Hospital”) (See supplementary Table 1 for the profile of the pathology department of the 5 hospitals). Abnormal liver function was determined if WBC exceeded the reference value (2 ~ 4 × 10^9^/L), namely, ALT > 40U/L or ASTT > 35U/L. Abnormal kidney function was determined if BUN > 7.6 mmol/L or Cr > 90 μmol/L. In addition, surveys regarding regular health examinations and self-administered questionnaires were conducted, which were administered by researchers who have received unified training to survey the subjects and explain the purpose. For medical technicians who were often exposed to a variety of hazardous chemicals, the questionnaire consisted of items related to smell disorder (such as hyposmia, anosmia, allergy, inversion, or phantom smell), eye discomfort (itchy, red, or dry eyes), and upper airway irritation (sneezing, dry or runny nose, and itchy throat). The medical examiners made the judgement based on the aforementioned criteria. This study began the status surveys, measure development, and intervention formulation in 2021, with 2020 as “before comprehensive intervention” and 2022 as “after comprehensive intervention”. In the year 2020, prior to the implementation of interventions, there was a total of 20 pathological technicians, 6 cutting-up benches, 35 fume cupboards, and 6 ventilated and expellant drying cabinets across the five hospitals. By 2022, one employee had retired, and three new pathological technicians were hired. As a result, there were a total of 22 pathological technicians, 10 cutting-up benches, 41 fume cupboards, and 10 ventilated and expellant drying cabinets. Data for the evaluation of conditions before and after the interventions were collected on 12 occasions throughout the year. These assessments were conducted consistently on the afternoon of the first Friday of each month, with fixed sampling sites. During this period, the workload increased from 121,196 cases in 2020 to 144,447 cases in 2022. Notably, the temperature and humidity levels within the pathology department remained within the specified range, with temperature ranging from 18 to 30℃ and humidity maintained between 45 and 75% (refer to Supplementary Table 1 for details).

### Determination methods

Air sampling was conducted in the technical room and the diagnostic room on the afternoon of the first Friday of each month. Over this period, a total of 360 specimens were collected, calculated as 12 months per year, 2 rooms, 3 years, and 5 samples per event. The sampling was carried out in accordance with *Sampling Specification for Monitoring and Sampling of Toxic Substances in the Air in the Workplaces* (GBZ 159–2004) [16]; The concentration of benzene was measured in accordance with the recommended gas phase liquid chromatography in the *Determination of Toxic Substances in the Air in the Workplaces -Aromatic Hydrocarbon Compounds* (GBZ/T 160.42—2007) [17]; the concentration of formaldehyde was measured in accordance with the recommended phenol reagent spectrophotometry in the *Determination of Toxic Substances in the Air in the Workplaces -Aliphatic Aldehydes Compounds* (GBZ/T 160.54—2007) [18]. Zhejiang Huabiao Testing Technology Co., Ltd. was commissioned to conduct the monitoring. The full-automatic atmospheric sampler (MH1200-B) by Qingdao Minhop Electronic Instrument Co., Ltd. was used for air sampling. The sampling was performed at two sampling sites (technical room and diagnostic room) of each hospital. The SP-752PC UV–visible spectrophotometer by Shanghai Spectrum Instruments Co., Ltd. and 7890 gas chromatograph (FID detector, U.S. Agilent Corporation) were used for detecting formaldehyde and benzene, respectively.

Sampling was performed according to the guidelines of *Sampling Specification for Monitoring Toxic Substances in Workplace Air *(GBZ 159–2004) [16]. Short-term sampling at one fixed time point at the workplace was performed for formaldehyde detection, and long-term individual sampling was performed for benzene detection for medical staff in different positions, both under normal working conditions. Benzene and formaldehyde concentrations were determined according to the guidelines of *Determination of Toxic Substances in Workplace Air-Aromatic Hydrocarbons *(GBZ/T 160.42–2007) [17] and *Determination of Toxic Substances in Workplace Air-Aliphatic Aldehydes *(GBZ/T 160.54–2007) [18], respectively.

### EPA risk assessment paradigm

#### Assessment of carcinogenic risks

Carcinogenic risk resulting from inhalation was calculated using the following formula: risk = lifetime average daily dose (LADD)_inh_ × inhalation unit risk (IUR); LADD_inh_ = (concentration [C] × exposure frequency [EF] × exposure duration [ED] × ET)/lifetime [LT] (Table 1) [7]. Air pollutants were considered to have a low risk for cancer if their lifetime carcinogenic risk coefficient was less than 1 × 10^–6^; a cancer risk was considered to exist if the risk coefficient was 1 × 10^–6^- 1 × 10^–4^,and there was a high risk if greater than 1 × 10^–4^.

**Table 1:** Inhalation exposure parameters

**Parameter**	**Description**
RISK	Carcinogenic risk coefficient
HQ	Noncarcinogenic hazard quotient
ADD_inh_	Average daily dose via inhalation (μg/m^3^)
LADD_inh_	Lifetime average daily dose via inhalation (μg/m^3^)
C	Pollutant concentration (μg/m^3^)
ED	Exposure duration (years), calculated based on the assumption of starting work between the ages of 24 to 28 and retiring at 55 for female workers and 60 for male workers, resulting in an estimated EDof 30 years
EF	Exposure frequency (d/year), calculated considering a standard work year of 52 weeks, with 5 working days per week, accounting for 11 legal holidays, and incorporating some overtime
ET	Exposure time (h/d), regarded as 8 h, which represents the standard daily working hours
AT	Average time (hours), 30 years × 365 days × 24 h = 262,800 h
LT	Lifetime (h) (life expectancy of the household population in Hangzhou, Zhejiang, China, in 2022: 83.63 years)
IUR	Inhalation unit risk (μg/m^3^)^−1^ (CDC & ATSDR: formaldehyde: 13.0 × 10^–6^; benzene: 2.2—7.8 × 10^–6^)
RfC	Inhalation reference dose (μg/m^3^) (CDC ATSDR: formaldehyde: 9.83; benzene: 30)

*Abbreviations: HQ* Hazard quotient, *ADD*_*inh*_ Average daily dose via inhalation, *LADD*_*inh*_ Lifetime average daily dose via inhalation, *AT* Average time, *LT* Lifetime, *IUR* Inhalation unit risk, *C* Mass concentration of contaminant, *EF* Exposure frequency, *ED* Exposure duration, *ET* Exposure time, *RfC* Reference concentration, *CDC* Centers for Disease Control and Prevention, *ATSDR* Agency for Toxic Substances and Disease Registry

#### Assessment of noncarcinogenic risks

Noncarcinogenic risk resulting from inhalation was calculated using the following formula: hazard quotient (HQ) = average daily dose via inhalation (ADD_inh_)/reference concentration (RfC); ADD_inh_ = (C × EF × ED × Exposure frequency[ET])/Average time[AT] (Table 1) [7]. The reference value of HQ was 1, with HQ ≥ 1 indicating a high noncarcinogenic risk and < 1 indicating a low noncarcinogenic risk.

### Problems before comprehensive interventions and comprehensive intervention measures

Given the different sizes of the pathology department of the five primary hospitals and the quality of medical staff, different problems existed. Through in-depth research, the essence of the problems was identified and personalized, and comprehensive interventions were developed for symptomatic and comprehensive rectification (Table 2).

**Table 2:** Problems with the prevention of occupational hazards in the technical room of the pathology department and the comprehensive interventions

**Problems**		**Interventions**	**Integrated measures**
Reagent	1. In the conventional fixation with formaldehyde reagent, the amount of formaldehyde used was more subjective 2. Chemical reagents such as benzene and its derivatives should be used for dehydration, embedding, slicing, and staining	1. The determination of the minimum formaldehyde amount for specimens of varying volumes involved conducting tests where formaldehyde was used in quantities ranging from 3 to 7 times the volume of specimens. This testing aimed to establish the minimum formaldehyde requirement for achieving the same quality in fixed tissue slices. The ultimate goal of this process is to set a standardized protocol that reduces the overall amount of formaldehyde needed for tissue fixation, thereby promoting resource efficiency and minimizing chemical usage in the laboratory 2. The dewaxing solution (main ingredients: solvent oil and chaotropic agent) by Ningbo Tongsheng Bio-technology Co., Ltd. was used instead of traditional benzene and derivative reagents for dehydration. A transparent liquid (main ingredients: solvent oil and esters) by Ningbo Tongsheng Bio-technology Co., Ltd.) was used instead of the neutral gum produced by Shanghai Specimen and Model Factory for sealing 3. The appropriate change time of stationary liquid was determined after tests (the formaldehyde stationary liquid was changed for every 10 days, 14 days and 20 days, respectively. Then, the appropriate frequency of change for the same mass of qualified slices was determined). The change frequency of the stationary liquid formaldehyde was changed from the original 10 days to 14 days, so as to reduce the times of change of formaldehyde and lowers the costs	Control of pollutant sources
Protective equipment	1. Face shields were reused after simple disinfection, without an eye mask 2. Some protective equipment was not worn properly	1. Disposable face shields were purchased to eliminate repeated use 2. The personnel of the nosocomial infection department were invited to provide uniform training for the staff of the pathology department, and everyone had to pass examinations. The protective equipment had to be worn properly in accordance with the Technical Specifications for Hospital Quarantine	
Facilities and equipment	1. Some departments fell short of the standard in terms of area, with unreasonable layouts 2. Some biosafety cabinets showed signs of aging; the ventilation capacity of some laboratories’ ventilation systems exhibited significant deterioiration; and some hospitals lacked fresh air systems	1. The area of the pathology department had to be increased: (1) The District MCH Hospital increased the area by 150m^2^, where the area of technical room was increased by 100m^2^; (2) The Second District Hospital increased this area by 50 m^2^, where the area of technical room was increased by 30m^2^; (3) The District TCM Hospital increased this area by 70m^2^, where the area of technical room was increased by 60m^2^; (4) The First District Hospital and the District Orthopedic Hospital did not increase this area, but optimized the layout 2. Four cutting-up benches had to be added (J-A1) (1 set for the First District Hospital, the Second District Hospital, the District MCH Hospital and the District TCM Hospital, respectively), and 6 fume cupboards (fume cupboards) (1 set for the First District Hospital, 1 set for the Second District Hospital, 2 sets for the District MCH Hospital, 1 set for the District TCM Hospital, and 1 set for the District Orthopedic Hospital) which were manufactured by JiaXingJinJing Metal Products Co., Ltd 3. Four ventilated and expellant drying cabinets had to be added (1 set for the First District Hospital, the Second District Hospital, the District MCH Hospital and the District TCM Hospital, respectively) which were manufactured by JiaXingJinJing Metal Products Co., Ltd	Enhance of pollutant discharge
Managers of the hospital and the pathology department	1. Insufficient attention was being paid to the occupational hazards of pathological technicians; and no sufficient efforts had been made in terms of inspection and rectification regarding protection and related monitoring 2. The training pertaining to occupational safety protection and the control of nosocomial infections lacked a systematic approach 3. There was lack of innovative and systematic management methods	1. Establishment of a technical personnel training system: The staff in the pathology department are subject to relevant training and assessment every month (the assessment results are divided into: excellent, passed and failed). The personal performance for the month is adjusted based on the assessment results (A reward of 300 yuan is granted for those who achieved excellent assessment results, and a fine of 300 yuan is imposed against those who failed the assessment) 2. Innovation of management in the technical room: The construction and management were standardized. The department was structured into sub-units, namely the Conventional Group, Immunohistochemistry Group, and Special Staining Group, based on practical requirements. Concurrently, a dual-track training program and qualification certification process were instituted for employees. In each quarter, experts at provincial hospitals will be invited to conduct on-site inspection and systematic training, so that the theories and practice are organically combined 3. Visits to pathology departments of affiliate hospitals to learn “7S” excellent management practices: Drawing from this advanced experience and aligning it with the department’s specific needs, the entire staff actively engages in a brainstorming session focusing on “7S” management principles. To ensure ongoing improvement, monthly evaluations of departmental sub-units are conducted, with these appraisal results serving as crucial benchmarks for the year-end assessment and performance evaluation process	Refined management
Medical workers	1. The awareness of occupational hazards and their protection was found to be considerably weak; and the protection habits were poor 2. Psychological stress was high, which resulted in psychological problems and affected physical health 3. Insufficient staff led to prolonged working hours and high intensity	1. Occupational hazards are included into the course for new employees in the department. Cases of occupational hazards are systematically compiled and shared within the department every six months. This initiative serves to elevate employees’ consciousness regarding the importance of safeguarding themselves against occupational risks 2. The acquisition of professional knowledge is bolstered while concurrently fostering psychological adaptability and resilience. Maintaining a positive and optimistic mindset is key, serving as a foundation to fortify self-confidence 3. Each hospital’s pathology department has repurposed well-ventilated, sterile rooms into designated male and female duty rooms. This modification allows employees to take breaks during lunchtime, weekends, and holidays, thus aiding in the preservation of their physical vitality	

Remarks: 1. First District Hospital: The hospital first replaced traditional chemical reagents with recycled environmentally friendly reagents such as dewaxed solution and transparent liquid, and then promoted the practice after the effect was proven

2. Second District Hospital: The hospital first tried to reduce the use of fixed formaldehyde. The approach involved investigating the feasibility of employing an amount equivalent to four times the specimen volume for specimen fixation, all the while ensuring the maintenance of slice quality. Subsequently, this practice was extended and implemented throughout the entire district

3. District MCH Hospital: Taking the relocation of the inpatient building as an opportunity, the hospital invited authoritative experts to design the pathology department, and purchased reasonable equipment. In addition, the provincial pathology center was invited to conduct on-site acceptance, and irelated remedial measures were also implemented in other hospitals

4. District TCM Hospital: The hospital initially implemented “7S” management principles, consistently conducting reviews and assessments. The insights gained from this experience were then shared with pathology departments in other hospitals for their reference and potential improvement

5. District Orthopedic Hospital: The hospital initially introduced a home-based standby system for employees residing near the hospital during holidays. Non-essential employees were temporarily relocated from the department. Following the trial’s completion, the outcomes and findings were shared with the pathology departments of other hospitals for their consideration and potential adoption

### Statistical analysis

Determination data were manually entered into an Excel database, and a calculation program was established according to the formula to determine the occupational hazard risk levels of benzene and formaldehyde among the medical staff. SPSS 26.0 software (IBM, Armonk, NY, USA) was used for data processing. The normality of the data distribution was evaluated using the Shapiro–Wilk test and expressed as mean ± standard deviation. An independent sample *t*-test was used to analyze the difference between both groups. The chi-square test was used to compare the rates between both groups *P* < 0.05 was considered statistically significant.

## Results

### Demographics of the technicians and clinical medical staff at the pathology department

Regarding age, sex, title, and educational background, there was no statistical difference between both groups (*P* > 0.05, Table 3).

**Table 3:** Demographics of technicians and clinical medical staff in the pathology department

**Variables**	**Case (n)**	**Age (years)**	**Sex (m/f, n)**	**Title (n)**			**Educational background (n)**		
				**Primary**	**Intermediate**	**Advanced**	**College**	**Undergraduate**	**Graduate**
Technician	22	36.82 ± 10.03	7/15	5	15	2	4	16	2
Clinical medical staff	24	36.29 ± 10.00	7/17	6	15	3	3	18	3
Statistical values	-	T = 0.178	Z = -0.193	Z = -0.600			Z = -0.117		
*P* value	-	0.859	0.847	0.548			0.907		

#### Effect of integrated measures adopted during intervention

**Fig. 1:**
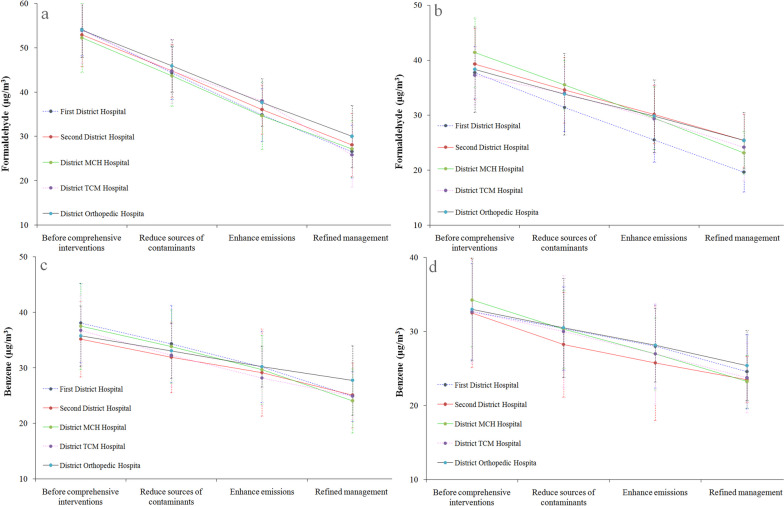
The influences of different interventions on formaldehyde and benzene in the technical room and the diagnostic room of the pathology department

The three comprehensive interventions were sequentially implemented. The first step involved reducing the sources of pollutants, leading to a reduction in formaldehyde and benzene levels in the technical and diagnostic rooms of the pathology departments in all five hospitals. Building upon this reduction, the second intervention focused on enhancing the removal of pollutant sources. This further decreased the formaldehyde and benzene concentrations in these rooms. Lastly, the third intervention introduced refined management practices, resulting in an additional reduction in formaldehyde and benzene levels within the technical and diagnostic rooms of the pathology departments across all five hospitals (See Fig. 1).

### Determination results of occupational hazards

After an on-site hygiene investigation, the determination results of formaldehyde and benzene concentrations at the pathology department of the five primary hospitals showed that10 testing sites met the indoor air quality standards (*Indoor Air Quality Standards *[GBGB/T18883-2002]). However, benzene used at a few sites was slightly higher than the standards of *Indoor Air Quality Standards* (GBGB/T18883-2002). Before comprehensive interventions, the formaldehyde concentrations in the technical rooms were higher than in the diagnostic rooms (*P* < 0.05). The 10 test sites in the five primary hospitals had higher formaldehyde and benzene concentrations before than after comprehensive interventions, with the difference being statistically significant (*P* < 0.05). However, after comprehensive interventions, all the testing sites met the requirements of *Indoor Air Quality Standards* (GBGB/T18883-2022). Conversely, except for the First District Hospital, there appeared to be no significant difference in formaldehyde and benzene concentrations in the remaining four hospitals after comprehensive interventions (*P* > 0.05, Table 4).

**Table 4:** Detection results of benzene and formaldehyde concentrations in the pathology department of the five primary hospitals

**Workplace**	**Formaldehyde (μg/m**^**3**^**)**				**Benzene (μg/m**^**3**^**)**			
	**Before comprehensive interventions**	**After comprehensive interventions**	**t value**	***P* value**	**Before comprehensive interventions**	**After comprehensive interventions**	**t value**	***P* value**
First District Hospital								
Technical room	54.167 ± 6.686	26.583 ± 4.944	11.491	< 0.001	38.0833 ± 7.115	24.917 ± 4.522	4.848	< 0.001
Diagnostic room	37.750 ± 4.731	19.667 ± 3.601	10.535	< 0.001	32.667 ± 6.485	24.583 ± 4.999	3.420	0.002
*t* value	6.943	3.917	-	-	1.7888	0.171	-	-
*P* value	< 0.001	0.001	-	-	0.088	0.866	-	-
Second District Hospital								
Technical room	52.917 ± 7.128	28.083 ± 7.128	7.830	< 0.001	35.167 ± 6.793	25.0833 ± 5.584	3.174	0.004
Diagnostic room	39.333 ± 6.443	25.417 ± 4.814	5.994	< 0.001	32.500 ± 7.342	23.500 ± 3.119	2.754	0.012
*t* value	4.897	0.958	-	-	0.610	0.827	-	-
*P* value	< 0.001	0.349	-	-	0.548	0.417	-	-
District MCH Hospital								
Technical room	52.250 ± 7.736	27.167 ± 6.365	7.896	< 0.001	37.500 ± 7.752	24.0833 ± 5.760	4.813	< 0.001
Diagnostic room	41.417 ± 6.288	23.167 ± 3.857	8.570	< 0.001	34.250 ± 6.312	23.250 ± 3.571	5.254	< 0.001
*t* value	3.424	1.862	-	-	1.126	0.426	-	-
*P* value	0.02	0.076	-	-	0.272	0.674	-	-
District TCM Hospital								
Technical room	53.917 ± 7.304	25.833 ± 7.209	7.971	< 0.001	36.750 ± 6.254	24.91 ± 4.209	5.437	< 0.001
Diagnostic room	37.250 ± 5.259	24.167 ± 6.028	4.195	< 0.001	32.667 ± 6.959	23.750 ± 4.693	3.340	0.003
*t* value	4.992	0.503	-	-	1.357	0.641	-	-
*P* value	< 0.001	0.620	-	-	0.188	0.528	-	-
District Orthopedic Hospital								
Technical room	54.000 ± 6.164	30.417 ± 6.402	7.405	< 0.001	35.750 ± 5.446	27.750 ± 6.240	3.346	0.003
Diagnostic room	38.333 ± 7.785	25.417 ± 5.143	4796	< 0.001	33.000 ± 6.954	25.417 ± 4.757	2.840	0.010
*t* value	5.465	1.650	-	-	0.990	1.030	-	-
*P* value	< 0.001	0.113	-	-	0.333	0.314	-	-

2021 indoor air quality testing (before comprehensive interventions)was in accordance with the guidelines of *Indoor Air Quality Standards*(GB/T18883-2002): formaldehyde ≤ 0.10mg/m^3^ and benzene ≤ 0.11mg/m^3^; 2022 indoor air quality testing (after comprehensive interventions)was in accordance with the guidelines of *Indoor Air Quality Standards*(GB/T18883-2022): formaldehyde ≤ 0.08mg/m^3^ and benzene ≤ 0.03mg/m^3^. t value: statistic value of t-test

*Abbreviations: MCH* Maternal and Child Health, *TCM* Traditional Chinese Medicine

### Carcinogenic risk

Medium risk values were observed with occupational exposure to benzene and formaldehyde among staff in both rooms before and after comprehensive interventions, although higher before than after comprehensive interventions. Furthermore, the staff in thetechnical rooms showed higher risk values than those in the diagnostic rooms before and after comprehensive interventions (Tables 5 and 6) (See Supplementary Tables 2 and 3 for the detailed calculation process).

**Table 5:** Estimation of carcinogenic and noncarcinogenic risk based on area exposure to formaldehyde in hospital laboratories over a daily work shift of 8 h

**Hospital**	**Carcinogenic risks of formaldehyde exposure among staff in the technical room (× 10**^**–4**^**)**			**Carcinogenic risks of formaldehyde exposure among staff in the diagnostic room (× 10**^**–4**^**)**			**Noncarcinogenic risks of formaldehyde exposure among staff in the technical room**			**Noncarcinogenic risks of formaldehyde exposure among staff in the diagnostic room**		
	**Before comprehensive interventions**	**After comprehensive interventions**	***P* value**	**Before comprehensive interventions**	**After comprehensive interventions**	***P* value**	**Before comprehensive interventions**	**After comprehensive interventions**	***P* value**	**Before comprehensive interventions**	**After comprehensive interventions**	***P* value**
First District Hospital	0.579 ± 0.071	0.284 ± 0.053	< 0.001	0.404 ± 0.051	0.210 ± 0.038	< 0.001	1.263 ± 0.156	0.620 ± 0.115	< 0.001	0.880 ± 0.110	0.459 ± 0.084	< 0.001
Second District Hospital	0.566 ± 0.076	0.300 ± 0.089	< 0.001	0.421 ± 0.069	0.272 ± 0.051	< 0.001	1.234 ± 0.166	0.655 ± 0.195	< 0.001	0.917 ± 0.150	0.593 ± 0.092	< 0.001
District MCH Hospital	0.559 ± 0.096	0.290 ± 0.068	< 0.001	0.443 ± 0.067	0.248 ± 0.041	< 0.001	1.218 ± 0.209	0.634 ± 0.148	< 0.001	0.966 ± 0.146	0.540 ± 0.090	< 0.001
District TCM Hospital	0.576 ± 0.078	0.276 ± 0.055	< 0.001	0.398 ± 0.075	0.258 ± 0.064	< 0.001	1.257 ± 0.170	0.602 ± 0.228	< 0.001	0.869 ± 0.156	0.564 ± 0.094	< 0.001
District Orthopedic Hospital	0.577 ± 0.066	0.325 ± 0.085	< 0.001	0.410 ± 0.073	0.272 ± 0.055	< 0.001	1.259 ± 0.144	0.709 ± 0.213	< 0.001	0.894 ± 0.162	0.593 ± 0.102	< 0.001
Mean 5 ± SD	0.571 ± 0.076	0.295 ± 0.084	< 0.001	0.414 ± 0.074	0.252 ± 0.054	< 0.001	2.246 ± 0.166	0.644 ± 0.151	< 0.001	0.905 ± 0161	0.550 ± 0.106	< 0.001

**Table 6:** Estimation of carcinogenic and noncarcinogenic risk based on area exposure to benzene in hospital laboratories over a daily work shift of 8 h

**Hospital**	**Carcinogenic risks of benzene exposure among staff in the technical room (× 10**^**–4**^**)**			**Carcinogenic risks of benzene exposure among staff in the diagnostic room (× 10**^**–4**^**)**			**Noncarcinogenic risks of benzene exposure among staff in the technical room**			**Noncarcinogenic risks of benzene exposure among staff in the diagnostic room**		
	**Before comprehensive interventions**	**After comprehensive interventions**	***P* value**	**Before comprehensive interventions**	**After comprehensive interventions**	***P* value**	**Before comprehensive interventions**	**After comprehensive interventions**	***P* value**	**Before comprehensive interventions**	**After comprehensive interventions**	***P* value**
First District Hospital	0.244 ± 0.053	0.160 ± 0.029	< 0.001	0.210 ± 0.042	0.158 ± 0.032	0.002	0.291 ± 0.063	0.190 ± 0.035	< 0.001	0.250 ± 0.050	0.189 ± 0.038	0.002
Second District Hospital	0.225 ± 0.058	0.161 ± 0.038	0.004	0.208 ± 0.070	0.151 ± 0.020	0.012	0.267 ± 0.070	0.192 ± 0.045	0.004	0.248 ± 0.083	0.180 ± 0.024	0.012
District MCH Hospital	0.241 ± 0.049	0.155 ± 0.037	< 0.001	0.220 ± 0.041	0.149 ± 0.023	< 0.001	0.287 ± 0.059	0.184 ± 0.044	< 0.001	0.262 ± 0.048	0.178 ± 0.027	< 0.001
District TCM Hospital	0.236 ± 0.040	0.160 ± 0.027	< 0.001	0.210 ± 0.052	0.152 ± 0.030	0.003	0.281 ± 0.048	0.190 ± 0.032	< 0.001	0.250 ± 0.062	0.182 ± 0.036	0.003
District Orthopedic Hospital	0.230 ± 0.035	0.178 ± 0.400	0.003	0.212 ± 0.051	0.163 ± 0.031	0.010	0.273 ± 0.042	0.212 ± 0.047	0.003	0.252 ± 0.061	0.194 ± 0.036	< 0.010
Mean 5 ± SD	0.235 ± 0.047	0.163 ± 0.034	< 0.001	0.212 ± 0.050	0.155 ± 0.027	< 0.001	0.280 ± 0.056	0.194 ± 0.040	< 0.001	0.252 ± 0.059	0.184 ± 0.030	< 0.001

### Noncarcinogenic risk

Before the comprehensive interventions, the HQ (Hazard Quotient) values for occupational exposure to formaldehyde in the technical rooms exceeded 1, indicating a higher noncarcinogenic risk associated with exposure. However, in the diagnostic rooms before the interventions, the HQ values for occupational exposure to formaldehyde were below 1, signifying a lower noncarcinogenic risk. The HQ values for occupational exposure to benzene and formaldehyde were less than 1 in both rooms after comprehensive interventions, with lower noncarcinogenic risks. Before comprehensive interventions, the HQ values of the occupational exposure to benzene and formaldehyde in the staff of the technical and diagnostic rooms were higher than those after comprehensive interventions. The staff in the technical rooms had higher HQ values before and after comprehensive interventions than those in the diagnostic rooms (Tables 5 and 6) (See Supplementary Tables 4 and 5 for the detailed calculation process).

### Health status of the technicians and clinical medical staff at the pathology department

**Fig. 2:**
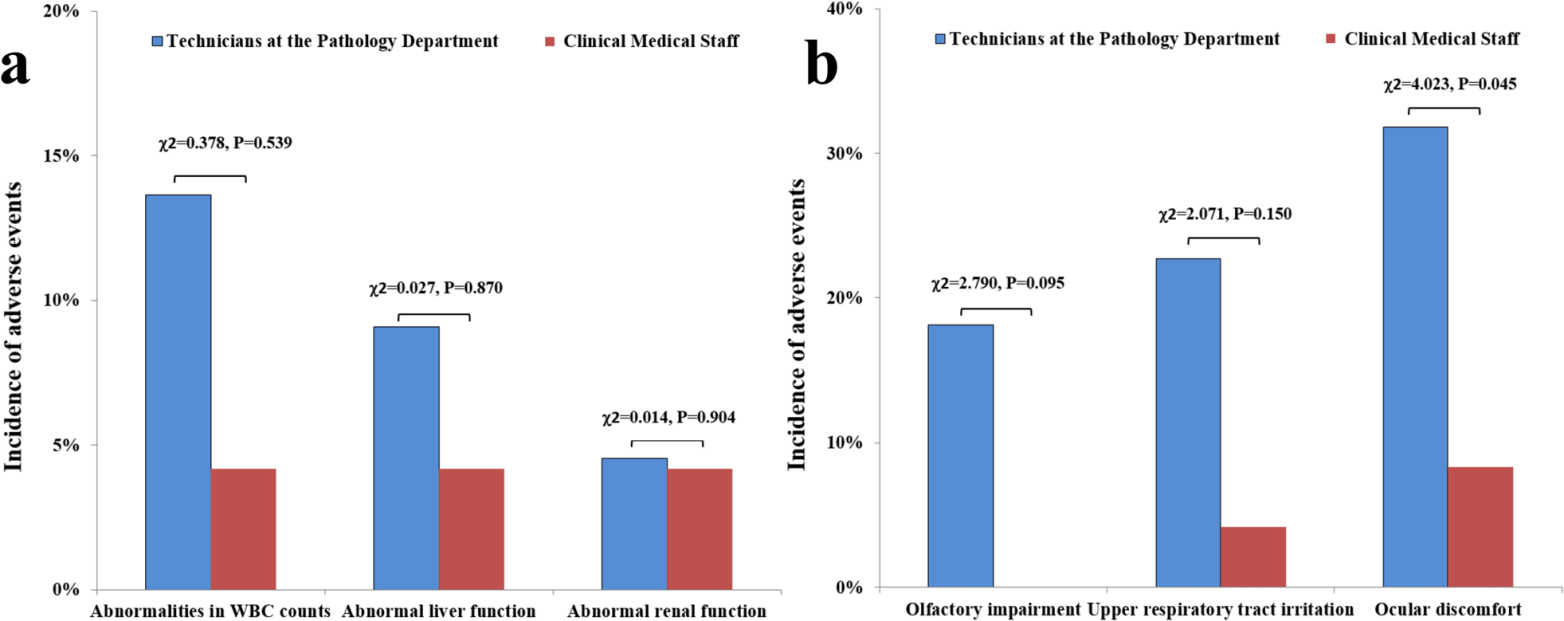
Comparison of the health status of the technicians and clinical medical staff at the pathology department. **a** Abnormal white blood cell count and abnormal liver and kidney functions **b** Smell disorder, eye discomfort, and upper airway irritation

Eye discomfort was higher among technicians than clinical medical staff (*P* < 0.05). However, the difference was insignificant in abnormal white blood cell (WBC) count, abnormal liver and kidney functions, smell disorder, eye discomfort, and upper airway irritation, despite the higher proportion of technicians compared to clinical medical staff (*P* > 0.05, Fig. 2).

### Quality evaluation of environmentally friendly reagents in rapid preparation of frozen sections

**Fig. 3:**
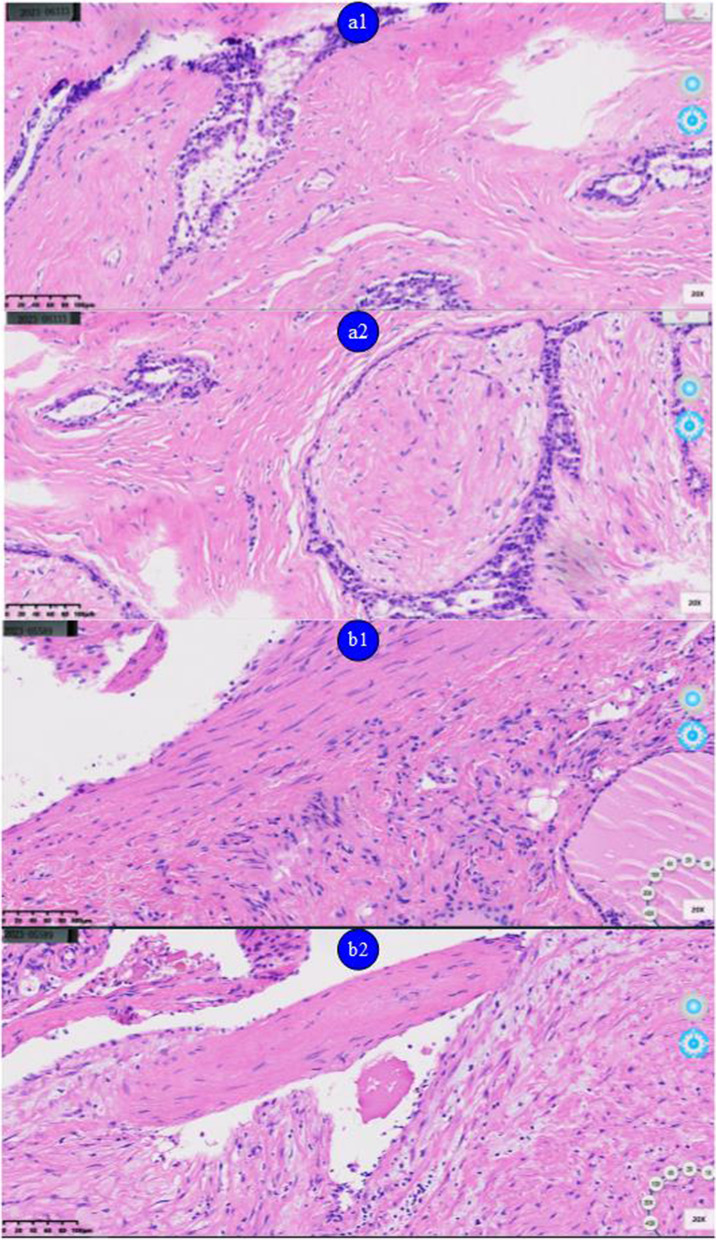
Hematoxylin–eosin staining of rapid frozen sections of thyroid and breast tissues using conventional and environmentally friendly reagents.  : Breast tissues using conventional reagents;  : Breast tissues environmentally friendly reagents;  : Thyroid using conventional reagents;  : Thyroid using environmentally friendly reagents

After rapid preparation of frozen tissue sections and staining of 20 cases of thyroid and breast tissues using conventional methods and environmentally friendly reagents, both methods were found to be effective, as observed under a microscope (Fig. 3).

## Discussion

Medical technicians in pathological laboratories are often exposed to a variety of harmful chemicals, such as benzene and formaldehyde, both of which are carcinogenic substances. Therefore, staff and hospital management should work in tandem to formulate and implement comprehensive interventions to ensure a safe occupational environment, reduce occupational hazards, and protect staff health, particularly staff in the pathology department in primary hospitals. Most studies have focused on whether benzene and formaldehyde concentrations in the technical room of a pathology department met the relevant standards and whether long-term exposure to certain concentrations of benzene and formaldehyde was a risk to human health in general. However, studies assessing the risk of benzene and formaldehyde exposure on the health of staff in the pathology departments are limited. Part A of the *Human Health Risk Assessment* recommended by the U.S.EPA and *Supplemental Guidance for Inhalation Risk Assessment* in Part F provide important technical references for risk assessment of occupational health hazards from indoor air. The assessment method takes into account the toxic hazards, exposure concentration, exposure time, and acute and chronic effects. Meanwhile, parameters such as RfC and IUR are supported by a large number of laboratories and epidemiological data, with few subjective components, and the assessment conclusions are scientific, rigorous, and credible [7, 13–15]. Therefore, a preliminary evaluation of benzene and formaldehyde concentrations in the technical rooms of the pathology department, combined with a human health risk assessment model, was conducted to explore their hazards to humans in-depth and provide a basis for further improving the preventive measures.

We first investigated the health status of the technicians and clinical medical staff in the pathology departments of five hospitals, and a statistical analysis was then conducted. The results showed that eye discomfort was the only finding that was statistically different between the two groups. In contrast, abnormal WBC count, abnormal liver and kidney functions, smell disorder, and upper airway irritation were predominantly higher among the technicians than the clinical medical staff, which was different from a previous study [19]. This numerical difference without statistical significance between both groups may be attributed to the study’s small sample size. Therefore, further studies with larger sample sizes are warranted.

The physical health of staff in the technical room was closely associated with chemical concentrations, such as formaldehyde and benzene, in indoor air. The high concentrations were due to the relatively independent technical and diagnostic rooms, a clear division of labor between the staff, and an overlap between them, coupled with large amounts of chemical reagents and greater chances of chemical exposure in the technical rooms. In this study, we found that formaldehyde and benzene concentrations in the technical rooms of the pathology department of the five primary hospitals in the district were higher than that in the diagnostic rooms. However, the formaldehyde concentrations detected in this study were slightly lower than those reported in previous reports [20]. After comprehensive interventions, including scientific planning, standardized operations, upgrading equipment, changing reagents, and improving self-protection, formaldehyde and benzene concentrations in the pathology department were significantly lowered. The exposure limits to formaldehyde and benzene according to the Indoor Air Quality Standards (GB/T18883-2002) were 0.10 mg/m3 and 0.11 mg/m3, respectively [21]. Whereas, these values were reduced to 0.08 mg/m^3^ and 0.03 mg/m^3^, respectively [22]. According to the U.S. ACGIH-TLV 2022, the safety levels of formaldehyde and benzene are 0.3 mg/m^3^ and 0.06 mg/m^3^, respectively. Although both values were lower than the domestic and foreign safety levels, and the concentration of benzene decreased significantly after comprehensive interventions (close to the national standard), interventions that further reduce their concentrations to a lower range are warranted.

We found that staff in the technical room occupationally exposed to benzene and formaldehyde had high carcinogenic and noncarcinogenic health risks. Although exposure to concentrations that meet authoritative standards may not cause significant damage in a short period, long-term retention of these substances in the body can pose a certain risk of carcinogenesis, The carcinogenic risk recommended by the EPA is not only positively correlated with the concentrations of formaldehyde and benzene but also with the life expectancy of the staff. As life expectancy increases worldwide, the risk of carcinogenesis maybe amplified. The current study revealed that the carcinogenic risk of formaldehyde and benzene in the technical and diagnostic rooms of the pathology department of five primary hospitals was remarkably reduced after comprehensive interventions, although medium risks remained. This indicated that long-term exposure to formaldehyde and benzene concentrations even within the acceptable limit still poses health risks to staff, which is in line with the findings of Li et al. [23] and Wang et al. [24]. The carcinogenic risk of formaldehyde and benzene among staff can only decrease when their concentrations in the workplace are reduced to lower or closer to the environmental concentration levels. Similarly, the noncarcinogenic risk of formaldehyde and benzene in the technical and diagnostic rooms of the pathology department of five primary hospitals was considerably reduced after comprehensive interventions, although it remained in the low-risk range before and after comprehensive interventions. These data indicated that the noncarcinogenic risks of formaldehyde and benzene were manageable. Compared with the conventional method of evaluating toxicity based on occupational exposure limits, risk assessment methods can comprehensively and quantitatively express the health risks of various populations exposed to toxins. If health risks were evaluated by health economics, the economic loss caused by various pollutants can be quantitatively estimated, which has an important guiding significance for occupational health management and decision-making. In addition, since the present study was conducted in primary hospitals, the limitation is that there was no testing of personal formaldehyde and benzene or monitoring of xylene. In the future, this will be rectified and the foregoing testing and monitoring will be included, so as to allow for the occupational hazards to be assessed in a more comprehensive manner.

Given the occupational health risks in the pathology department, active measures should be taken to reduce the occupational hazards of formaldehyde and benzene and minimize health risks. First, the use of chemical reagents, such as formaldehyde and benzene, should be reduced. Here, we attempted to replace traditional reagents with environmentally friendly reagents and completely change the environment of the pathology department. We also included rapid frozen tissue sections in the pilot project according to the department’s characteristics, and the results showed that the preparation quality of frozen tissue sections with environmentally friendly reagents was not significantly different from that with traditional methods and can meet the needs of daily clinical work. However, quality control, standardization, and toxicological experiments of environmentally friendly reagents have yet to be studied; in addition, the cost of using these agents is higher than those of traditional reagents. Therefore, there remains significant room for further innovation and development in completely applying environmentally friendly reagents instead of traditional reagents for the preparation of various tissue sections in the pathology department.

## Conclusion

Occupational health risks in the pathology department are a serious concern and need sufficient attention. Comprehensive interventions, including improving hardware facilities, increasing awareness and education on occupational hazards and protective measures among medical staff, optimizing hospital management, and reagent substitution can help reduce occupational exposure to harmful substances, such as formaldehyde and benzene. In addition, establishing a set of optimization programs that are suitable for individual pathology departments by drawing on advanced risk assessment models can effectively reduce indoor air pollution to environmental concentration levels, thereby preventing various health hazards.

### Supplementary Information

**Additional file 1:Table 1. **General comparison of the 5 hospitals before and after interventions.** Table 2. **Carcinogenic risks of formaldehyde exposure among staff in pathology department of the five primary hospitals.** Table 3. **Carcinogenic risks of benzene exposure among staff in the pathology department of the five primary hospitals.** Table 4. **Noncarcinogenic risks of formaldehyde exposure among staff in the pathology department of the five primary hospitals.** Table 5. **Noncarcinogenic risks of benzene exposure among staff in the pathology department of the five primary hospitals.

## Data Availability

We declared that materials described in the manuscript, including all relevant raw data, will be freely available to any scientist wishing to use them for non-commercial purposes, without breaching participant confidentiality.
