# Supplementary material for: Comprehensive interventions to reduce occupational hazards among medical staff in the pathology department of five primary hospitals
Source: BMC Public Health. 2023 Oct 31;23:2136. doi: 10.1186/s12889-023-16948-2 (PMC10617185; doi:10.1186/s12889-023-16948-2)
Supplement: Supplementary file 1 — Additional file 1:Table 1. General comparison of the 5 hospitals before and after interventions. Table 2. Carcinogenic risks of formaldehyde exposure among staff in pathology department of the five primary hospitals. Table 3. Carcinogenic risks of benzene exposure among staff in the pathology department of the five primary hospitals. Table 4. Noncarcinogenic risks of formaldehyde exposure among staff in the pathology department of the five primary hospitals. Table 5. Noncarcinogenic risks of benzene exposure among staff in the pathology department of the five primary hospitals. [file 12889_2023_16948_MOESM1_ESM.docx]

| **Table 1.** General comparison of the 5 hospitals before and after interventions | | | | | | | | | | |
| --- | --- | --- | --- | --- | --- | --- | --- | --- | --- | --- |
| Hospital | Year | Area of the pathology department (m^2^) | | | Pathologist (persons) | Quantity of equipment (pieces) | | | | Workload (cases/year) |
|  |  | Technical room | Diagnostic room | Total |  | Cutting-up bench | Fume cupboard | Ventilated and expellant drying cabinets | Total |  |
| First District Hospital | 2020 | 580 | 300 | 880 | 6 | 2 | 11 | 2 | 15 | 50086 |
|  | 2022 | 580 | 300 | 880 | 6 | 3 | 12 | 3 | 18 | 65374 |
| Second District Hospital | 2020 | 120 | 80 | 200 | 5 | 1 | 5 | 1 | 7 | 12608 |
|  | 2022 | 150 | 100 | 250 | 5 | 2 | 6 | 2 | 10 | 14130 |
| District MCH Hospital | 2020 | 100 | 50 | 150 | 3 | 1 | 9 | 1 | 11 | 27937 |
|  | 2022 | 200 | 100 | 300 | 4 | 2 | 11 | 2 | 15 | 30470 |
| District TCM Hospital | 2020 | 120 | 80 | 200 | 4 | 1 | 8 | 1 | 10 | 29334 |
|  | 2022 | 180 | 90 | 270 | 4 | 2 | 9 | 2 | 13 | 33185 |
| District Orthopedic Hospital | 2020 | 80 | 50 | 130 | 2 | 1 | 2 | 1 | 4 | 1231 |
|  | 2022 | 80 | 50 | 130 | 3 | 1 | 3 | 1 | 5 | 1288 |
| Total | 2020 | 1000 | 560 | 1560 | 20 | 6 | 35 | 6 | 47 | 121196 |
|  | 2022 | 1190 | 640 | 1830 | 22 | 10 | 41 | 10 | 61 | 144447 |
| Note: 2020: Before interventions; 2022: After interventions; cutting-up bench: J-A1 model (Jia Xing Jin Jing Metal Products Co., Ltd.): fume cupboard: J-E6 (Jia Xing Jin Jing Metal Products Co., Ltd.): ventilated and expellant drying cabinets: J-E5 (Jia Xing Jin Jing Metal Products Co., Ltd.) | | | | | | | | | | |

| **Table 2.** Carcinogenic risks of formaldehyde exposure among staff in pathology department of the five primary hospitals | | | | | | | | | | | | | | | |
| --- | --- | --- | --- | --- | --- | --- | --- | --- | --- | --- | --- | --- | --- | --- | --- |
| **Workplace** | | **Before comprehensive interventions** | | | | | | | **After comprehensive interventions** | | | | | | |
|  |  | **C (μg/m^3^)** | **EF (d/y)** | **ED (y)** | **ET (h/d)** | **LT (h)** | **IUR [× 10^-6^ (μg/m^3^)]^-1^** | **Risk (× 10^-4^)** | **C (μg/m^3^)** | **EF (d/y)** | **ED (y)** | **ET (h/d)** | **LT (h)** | **IUR [× 10^-6^,(μg/m^3^)]^-1^** | **Risk (× 10^-4^)** |
| First District Hospital | Technical room | 54.17 | 251 | 30 | 8 | 732599 | 13.0 | 0.579 | 26.58 | 251 | 30 | 8 | 732599 | 13.0 | 0.284 |
|  | Diagnostic room | 37.75 | 251 | 30 | 8 | 732599 | 13.0 | 0.404 | 19.67 | 251 | 30 | 8 | 732599 | 13.0 | 0.210 |
| Second District Hospital | Technical room | 52.97 | 251 | 30 | 8 | 732599 | 13.0 | 0.566 | 28.08 | 251 | 30 | 8 | 732599 | 13.0 | 0.300 |
|  | Diagnostic room | 39.33 | 251 | 30 | 8 | 732599 | 13.0 | 0.421 | 25.42 | 251 | 30 | 8 | 732599 | 13.0 | 0.272 |
| District MCH Hospital | Technical room | 52.25 | 251 | 30 | 8 | 732599 | 13.0 | 0.559 | 27.17 | 251 | 30 | 8 | 732599 | 13.0 | 0.290 |
|  | Diagnostic room | 41.42 | 251 | 30 | 8 | 732599 | 13.0 | 0.443 | 23.17 | 251 | 30 | 8 | 732599 | 13.0 | 0.248 |
| District TCM Hospital | Technical room | 53.92 | 251 | 30 | 8 | 732599 | 13.0 | 0.576 | 25.83 | 251 | 30 | 8 | 732599 | 13.0 | 0.276 |
|  | Diagnostic  room | 37.25 | 251 | 30 | 8 | 732599 | 13.0 | 0.398 | 24.17 | 251 | 30 | 8 | 732599 | 13.0 | 0.258 |
| District Orthopedic Hospital | Technical room | 54.00 | 251 | 30 | 8 | 732599 | 13.0 | 0.577 | 30.42 | 251 | 30 | 8 | 732599 | 13.0 | 0.321 |
|  | Diagnostic room | 38.33 | 251 | 30 | 8 | 732599 | 13.0 | 0.410 | 25.42 | 251 | 30 | 8 | 732599 | 13.0 | 0.272 |
| **Note:** Risk = C × EF × ED × ET × IUR/LT; Life expectancy of Hangzhou’s household population in 2022 was 83.63 years; High risk, Risk > 1 × 10^-4^; Medium risk, Risk: 1 × 10^-4^> Risk > 1 × 10^-6^ and Low risk: Risk < 1 × 10^-6^.  **Abbreviations:** C, pollutant concentration; EF, exposure frequency; ED, exposure duration; ET, exposure time; IUR, inhalation unit risk; LT, lifetime; MCH, Maternal and Child Health; TCM, Traditional Chinese Medicine. | | | | | | | | | | | | | | | |

| **Table 3.** Carcinogenic risks of benzene exposure among staff in the pathology department of the five primary hospitals | | | | | | | | | | | | | | | |
| --- | --- | --- | --- | --- | --- | --- | --- | --- | --- | --- | --- | --- | --- | --- | --- |
| **Workplace** | | **Before comprehensive interventions** | | | | | | | **After comprehensive interventions** | | | | | | |
|  |  | **C (μg/m^3^)** | **EF (d/y)** | **ED (y)** | **ET (h/d)** | **LT (h)** | **IUR [× 10^-6^ (μg/m^3^)]^-1^** | **Risk(× 10^-4^)** | **C (μg/m^3^)** | **EF (d/y)** | **ED (y)** | **ET (h/d)** | **LT (h)** | **IUR [× 10^-6^,(μg/m^3^)]^-1^** | **Risk(× 10^-4^)** |
| First District Hospital | Technical room | 38.09 | 251 | 30 | 8 | 732599 | 7.80 | 0.244 | 24.92 | 251 | 30 | 5.6 | 732599 | 7.8 | 0.160 |
|  | Diagnostic room | 32.67 | 251 | 30 | 8 | 732599 | 7.80 | 0.210 | 24.58 | 251 | 30 | 5.6 | 732599 | 7.8 | 0.158 |
| Second District Hospital | Technical room | 35.00 | 251 | 30 | 8 | 732599 | 7.80 | 0.225 | 25.08 | 251 | 30 | 5.6 | 732599 | 7.8 | 0.161 |
|  | Diagnostic room | 32.50 | 251 | 30 | 8 | 732599 | 7.80 | 0.208 | 23.50 | 251 | 30 | 5.6 | 732599 | 7.8 | 0.151 |
| District MCH Hospital | Technical room | 37.20 | 251 | 30 | 8 | 732599 | 7.80 | 0.241 | 24.08 | 251 | 30 | 5.6 | 732599 | 7.8 | 0.155 |
|  | Diagnostic room | 34.25 | 251 | 30 | 8 | 732599 | 7.80 | 0.220 | 23.25 | 251 | 30 | 5.6 | 732599 | 7.8 | 0.149 |
| District TCM Hospital | Technical room | 36.75 | 251 | 30 | 8 | 732599 | 7.80 | 0.236 | 24.92 | 251 | 30 | 5.6 | 732599 | 7.8 | 0.160 |
|  | Diagnostic room | 32.75 | 251 | 30 | 8 | 732599 | 7.80 | 0.210 | 23.75 | 251 | 30 | 5.6 | 732599 | 7.8 | 0.152 |
| District Orthopedic Hospital | Technical room | 35.75 | 251 | 30 | 8 | 732599 | 7.80 | 0.230 | 27.75 | 251 | 30 | 5.6 | 732599 | 7.8 | 0.178 |
|  | Diagnostic room | 33.00 | 251 | 30 | 8 | 732599 | 7.80 | 0.212 | 25.42 | 251 | 30 | 5.6 | 732599 | 7.8 | 0.163 |
| **Note:** Risk = C × EF × ED × ET × IUR/LT; Life expectancy of Hangzhou’s household population in 2022 was 83.63 years; High risk: Risk > 1 × 10^-4^; Medium risk: Risk1 × 10^-4^> Risk > 1 × 10^-6^ and Low risk: Risk < 1 × 10^-6^.  **Abbreviations:** C, pollutant concentration; EF, exposure frequency; ED, exposure duration; ET, exposure time; IUR, inhalation unit risk; LT, lifetime; MCH, Maternal and Child Health; TCM, Traditional Chinese Medicine. | | | | | | | | | | | | | | | |

| **Table 4.** Noncarcinogenic risks of formaldehyde exposure among staff in the pathology department of the five primary hospitals | | | | | | | | | | | | | | | |
| --- | --- | --- | --- | --- | --- | --- | --- | --- | --- | --- | --- | --- | --- | --- | --- |
| **Workplace** | | **Before comprehensive interventions** | | | | | | | **After comprehensive interventions** | | | | | | |
|  |  | **C (μg/m^3^)** | **EF (d/y)** | **ED (y)** | **ET (h/d)** | **AT (h)** | **RfC(μg/m^3^)** | **HQ** | **C (μg/m^3^)** | **EF (d/y)** | **ED (y)** | **ET (h/d)** | **AT (h)** | **RfC(μg/m^3^)** | **HQ** |
| First District Hospital | Technical room | 54.17 | 251 | 30 | 8 | 262800 | 9.83 | 1.263 | 26.58 | 251 | 30 | 8 | 262800 | 9.83 | 0.620 |
|  | Diagnostic room | 37.75 | 251 | 30 | 8 | 262800 | 9.83 | 0.880 | 19.67 | 251 | 30 | 8 | 262800 | 9.83 | 0.459 |
| Second District Hospital | Technical room | 52.97 | 251 | 30 | 8 | 262800 | 9.83 | 1.234 | 28.08 | 251 | 30 | 8 | 262800 | 9.83 | 0.655 |
|  | Diagnostic room | 39.33 | 251 | 30 | 8 | 262800 | 9.83 | 0.917 | 25.42 | 251 | 30 | 8 | 262800 | 9.83 | 0.593 |
| District MCH Hospital | Technical room | 52.25 | 251 | 30 | 8 | 262800 | 9.83 | 1.218 | 27.17 | 251 | 30 | 8 | 262800 | 9.83 | 0.633 |
|  | Diagnostic room | 41.42 | 251 | 30 | 8 | 262800 | 9.83 | 0.966 | 23.17 | 251 | 30 | 8 | 262800 | 9.83 | 0.540 |
| District TCM Hospital | Technical room | 53.92 | 251 | 30 | 8 | 262800 | 9.83 | 1.257 | 25.83 | 251 | 30 | 8 | 262800 | 9.83 | 0.602 |
|  | Diagnostic room | 37.25 | 251 | 30 | 8 | 262800 | 9.83 | 0.869 | 24.17 | 251 | 30 | 8 | 262800 | 9.83 | 0.564 |
| District Orthopedic Hospital | Technical room | 54.00 | 251 | 30 | 8 | 262800 | 9.83 | 1.259 | 30.42 | 251 | 30 | 8 | 262800 | 9.83 | 0.700 |
|  | Diagnostic room | 38.33 | 251 | 30 | 8 | 262800 | 9.83 | 0.894 | 25.42 | 251 | 30 | 8 | 262800 | 9.83 | 0.593 |
| **Note:** HQ = C × EF × ED × ET × IUR/AT/RfC; HQ ≥1 indicates a higher non-carcinogenic risk; HQ <1 indicates a lower non-carcinogenic risk.  **Abbreviations:** HQ, hazard quotient; C, contaminant mass concentration; EF, exposure frequency; ED, exposure duration; ET, exposure time; AT, average time; RfC, reference concentration (time period for calculating the average exposure);MCH, Maternal and Child Health; TCM, Traditional Chinese Medicine. | | | | | | | | | | | | | | | |

| **Table 5.** Noncarcinogenic risks of benzene exposure among staff in the pathology department of the five primary hospitals | | | | | | | | | | | | | | | |
| --- | --- | --- | --- | --- | --- | --- | --- | --- | --- | --- | --- | --- | --- | --- | --- |
| **Workplace** | | **Before comprehensive interventions** | | | | | | | **After comprehensive interventions** | | | | | | |
|  |  | **C (μg/m^3^)** | **EF (d/y)** | **ED (y)** | **ET (h/d)** | **AT (h)** | **RfC(μg/m^3^)** | **HQ** | **C (μg/m^3^)** | **EF (d/y)** | **ED (y)** | **ET (h/d)** | **AT (h)** | **RfC(μg/m^3^)** | **HQ** |
| First District Hospital | Technical room | 38.09 | 251 | 30 | 8 | 262800 | 30 | 0.291 | 24.92 | 251 | 30 | 8 | 262800 | 30 | 0.190 |
|  | Diagnostic room | 32.67 | 251 | 30 | 8 | 262800 | 30 | 0.250 | 24.58 | 251 | 30 | 8 | 262800 | 30 | 0.189 |
| Second District Hospital | Technical room | 35.00 | 251 | 30 | 8 | 262800 | 30 | 0.267 | 25.08 | 251 | 30 | 8 | 262800 | 30 | 0.192 |
|  | Diagnostic room | 32.50 | 251 | 30 | 8 | 262800 | 30 | 0.248 | 23.50 | 251 | 30 | 8 | 262800 | 30 | 0.180 |
| District MCH Hospital | Technical room | 37.20 | 251 | 30 | 8 | 262800 | 30 | 0.287 | 24.08 | 251 | 30 | 8 | 262800 | 30 | 0.184 |
|  | Diagnostic room | 34.25 | 251 | 30 | 8 | 262800 | 30 | 0.262 | 23.25 | 251 | 30 | 8 | 262800 | 30 | 0.178 |
| District TCM Hospital | Technical room | 36.75 | 251 | 30 | 8 | 262800 | 30 | 0.281 | 24.92 | 251 | 30 | 8 | 262800 | 30 | 0.190 |
|  | Diagnostic room | 32.75 | 251 | 30 | 8 | 262800 | 30 | 0.250 | 23.75 | 251 | 30 | 8 | 262800 | 30 | 0.182 |
| District Orthopedic Hospital | Technical room | 35.75 | 251 | 30 | 8 | 262800 | 30 | 0.273 | 27.75 | 251 | 30 | 8 | 262800 | 30 | 0.212 |
|  | Diagnostic room | 33.00 | 251 | 30 | 8 | 262800 | 30 | 0.252 | 25.42 | 251 | 30 | 8 | 262800 | 30 | 0.194 |
| **Note:** HQ = C × EF × ED × ET × IUR/AT/RfC; HQ ≥1 indicates a higher non-carcinogenic risk; HQ < 1 indicates a lower non-carcinogenic risk.  **Abbreviations:** HQ, hazard quotient; C, contaminant mass concentration; EF, exposure frequency; ED, exposure duration; ET, exposure time; AT, average time; RfC, reference concentration (the time period for calculating the average exposure); MCH, Maternal and Child Health; TCM, Traditional Chinese Medicine. | | | | | | | | | | | | | | | |
